# The Carbon Isotope Composition of Epiphytes Depends Not Only on Their Layers, Life Forms, and Taxonomical Groups but Also on the Carbon and Nitrogen Indicators of Host Trees

**DOI:** 10.3390/plants12193500

**Published:** 2023-10-08

**Authors:** Alen K. Eskov, Tatiana G. Elumeeva, Vlad. D. Leonov, Sergey M. Tsurikov, Violetta A. Viktorova, Nikolay G. Prilepsky, Evgeny V. Abakumov

**Affiliations:** 1 Department of Plant Ecology and Geography, Moscow State University, Leninskie Gory 1, 119991 Moscow, Russia; 2 Severtsov Institute of Ecology and Evolution, Russian Academy of Sciences, 33 Leninskij Prosp., 119071 Moscow, Russia; 3 Tzitzin Main Botanical Garden, Russian Academy of Sciences, 127276 Moscow, Russia; 4 Department of Applied Ecology, Saint-Petersburg State University, 16 Line of VO 29, 199178 St. Petersburg, Russia; e_abakumov@mail.ru

**Keywords:** epiphytes, carbon isotope, stable isotopes, isotopic ecology, Vietnam

## Abstract

The carbon isotopic composition of plant tissues is a diagnostic feature of a number of physiological and ecological processes. The most important of which is the type of photosynthesis. In epiphytes, two peaks of δ^13^C values are known to correspond to C3 and CAM photosynthesis and some variants of transitional forms between them. But the diagnosis of δ^13^C may not be limited to the type of photosynthesis. This makes it necessary to study trends in the distribution of δ^13^C in a broader ecological context. In this study, we present trends in the distribution of δ^13^C epiphytes and other structurally dependent plants and their relationship with other isotopic and elemental parameters (δ^15^N, C%, N%, and C/N) and with life forms of epiphytes, taxonomic or vertical groups in crowns (synusia), and the parameters of the trees themselves. In all communities except for the moss forest, δ^13^C in epiphyte leaves was significantly higher (less negative) than in phorophyte leaves. In general, δ^13^C in epiphytes in mountain communities (pine forest and moss forest) was more negative than in other communities due to the absence of succulents with CAM. δ^13^C in the leaves of all epiphytes was negatively related to the percentage of carbon and δ^15^N in the leaves of the phorophyte. When considering the Gaussian distributions of δ^13^C with the method of modeling mixtures, we observe the unimodal, bimodal, and trimodal nature of the distribution.

## 1. Introduction

The carbon isotope composition of plant tissues is a diagnostic sign of a number of physiological and ecological processes. The most important of which is the type of photosynthesis. Its influence on the isotopic signature is explained by the varying degree of carbon isotope fractionation during the fixation of atmospheric carbon dioxide by plants [1,2]. According to this criterion, vascular plants can be divided into two main groups: on the one hand, C3 plants, and on the other hand, C4 and CAM plants [3].

For C3 plants, the range of δ^13^C variation varies from approximately −37 to −20‰, while for CAM and C4 plants, the approximate δ^13^C values are from −23 to −10‰ [4,5,6]. However, among epiphytes, the C4 type of photosynthesis has not been reported in any representative of this group, while CAM photosynthesis (Crassulacean acid metabolism—acid metabolism of Thistles) is quite widespread and well known. Overall, CAM has been reported in 370 genera of vascular plants, representing 38 families and representing almost 7% of all vascular plants [7]. The isotopic composition of vascular epiphytes from the New World [8,9,10,11,12,13,14,15], as well as Australia and New Guinea [16,17,18], has not yet been extensively studied. In contrast, the isotopic composition of vascular epiphytic floras of Southeast Asian floras is rather fragmentedly represented in modern studies [19,20,21], despite their high species richness. Thus, the carbon isotope composition among representatives of the group of vascular epiphytes can be represented as a bimodal distribution with maxima characteristic of C3 and CAM plants [3,12,22].

A recent large-scale study of δ^13^C values published for CAM/C3 vascular plant lineages, and new data presented for the succulents Mesembryanthemoideae (Aizoaceae) and Aeonieae (Crassulaceae), demonstrates that a bimodal distribution is not always present in all lineages. Mixed modeling also shows that the bimodal distribution of δ^13^C values in the full data set as well as in very well-selected bromeliads is best described using a combination of three rather than two Gaussian distributions with one intermediate cluster between the more obvious clusters of C3- and CAM-like values. Given these results, and the emerging unimodal distribution of δ^13^C values in Mesembryanthemoideae with a mean value close to −20‰, the authors conclude that the apparent continuum between CAM and C3 photosynthesis cautions against the use of a δ^13^C threshold in macroevolutionary studies [3].

It is well known the adaptation to water deficit is a key ecological property of CAM plants is [23]. Epiphytes with CAM gain some selective ecological advantage, as water scarcity is considered to be the main limiting factor for epiphytes [24]. Several patterns of CAM distribution are recognized for epiphytes. The first is that the percentage of CAM species increases with decreasing rainfall. This varies from 25% in the rainforests of New Guinea and Australia to 100% in the dry forests of Mexico [24]. The second is the decrease in the number of CAM species with altitude above sea level [25]. This correlation is caused, among other things, by a decrease in forest canopy height with increasing elevation. The forest canopy is generally lower in areas above 2000 m elevation, reducing the habitat available for epiphyte colonization [4]. And third, the proportion of epiphytes with CAM increases with trunk height in closed lowland forests [26], from 7% in the middle part of the canopy to 50% in the open areas of tree tops [9,27]. These trends have been detected mainly based on δ^13^C [28].

Plant species with a facultative type of CAM photosynthesis (C3-CAM) are plants capable of performing C3 photosynthesis and switching to the CAM pathway under certain stimuli such as water shortage, sudden change in light, and some others. For example, such an important model plant with facultative CAM, like *Mesembryanthemum crystallinum* L., does not show a clear distinction between (strong) CAM and the C3-CAM intermediate, as salinity, photosynthetic mode, and age have been found to affect its δ^13^C [29] as a result this species shows a range of δ^13^C from −16 to −30 ‰ that covers all types of photosynthesis [30,31,32].

This complicates the interpretation of the carbon isotopic signature as a criterion for the presence of CAM. Similarly, δ^13^C in the annual rings of trees [33,34,35] or prickles of cacti [36] can speak to the efficiency of moisture utilization or availability and, consequently, stress limiting photosynthesis. There are also a number of data showing the possibility of changes in carbon isotope composition not directly related to metabolism. For example, (1) there is the so-called “forest canopy effect”: in dense stands, the ^13^C concentration in plant leaves has a pronounced vertical gradient. It is minimal in the ground layer, which is associated with the peculiarities of photosynthesis under shading conditions and with the fixation of depleted ^13^C carbon dioxide released from soil and litter [37,38]. The forest canopy effect in some cases distinguishes “forest” carbon from “meadow” carbon and differentiates carbon fixed by plants of different tiers in the bodies of phytophagous and mycorrhizal fungi [39,40]. In (2) some cases, changes in the carbon isotopic composition of epiphyte tissues can be explained by mutualistic relationships with invertebrates, particularly ants. Studies of such relationships between the CAM epiphyte *Dischidia major* (Apocynaceae) and ants of *Philidris* sp. have shown that up to 39% of the carbon utilized by the epiphyte may come from ant-associated sources [41]. As a result of ants inhabiting the speciose sac-shaped leaves of *D. major* and feeding mainly on C3 plants, carbon dioxide within these epiphyte leaves becomes more enriched in the light isotope ^12^C. The δ^13^C value of leaves within which ants settle is significantly lower than that of uninhabited leaves and depends on the degree of leaf utilization by ants. And (3) finally, ^13^C enrichment may serve as a signature of partial mycoheterotrophy in autotrophic plants [42], although it is likely that this signature cannot be analogous in CAM [43].

Thus, the difficulty of unambiguously interpreting carbon isotopic signature signals requires consideration of the δ^13^C of epiphytes in a broader biological context, which has not been studied until now. We decided to go beyond considering δ^13^C solely as a marker of CAM and decided to investigate the ecological context in which the δ^13^C of epiphytes is embedded. This study, for the first time, presents trends in the δ^13^C distribution of leaves of epiphytes and other structurally dependent plants (semi-epiphytes and mistletoe semi-parasites) and their relationship with other isotopic and elemental parameters (δ^15^N, C%, N%, and C/N) and epiphyte life forms, taxonomic or vertical groups in crowns (synusia), and tree parameters on a large sample of epiphytes and phorophytes from six forest types of three national parks in South Vietnam, which is known as one of the least explored tropical regions.

We were motivated by the following research hypotheses: (1) δ^13^C will correlate with C and N parameters depending not only on the tier localization and taxonomic group of the epiphyte but also on its life form; (2) δ^13^C will be significantly correlated with C and N parameters of the phorophyte, due to the “forest canopy effect”; and (3) δ^15^N cannot be a predictor of δ^13^C changes in epiphytes due to the previously described δ^15^N correlation between phorophytes and epiphytes, which can be explained by the edificatory role of epiphytes.

## 2. Materials and Methods

### 2.1. Field Studies

Field research was conducted in Southern Vietnam in 2015–2016 in the following habitats. The ecosystem was an open savanna-like forest located on oligotrophic soils (Phu Quoc). Phu Quoc Island located in Gulf of Siam has subequatorial, humid climate with a short, relatively dry period in the winter (January–February). The studied areas of open savanna-like forests were located not far from both the islands coasts, around the line between 10°16′46″ N, 103°55′23″ E and 10°24′17″ N, 104°03′02″ E. The soils are very poor, oligotrophic with sandy-loam texture and low organic matter content in the fine earth [44]. Epiphytes were collected from 39 phorophyte individuals of the following species: *Melaleuca leucadendra* (Myrtaceae)*, Heritiera littoralis* (Malvaceae), *Dipterocarpus* aff. *tuberculatus* (Dipterocarpaceae), *Dillenia obovata* (Dilleniaceae), *Tristaniopsis merguensis* (Myrtaceae), and *Parinari anamensis* (Chrysobalanaceae). Trees were widely spaced and herb layer was poorly developed and alternated with large areas of bare sand soil surface. Many trees are infected by hemiparasites from the *Loranthaceae* and *Viscaceae* families. The community of vascular epiphytes was represented by 16 species from 5 families (3 of flowering plants and 2 of ferns). Additionally, representatives of two families of hemiparasites (3 species) were present. Myrmecophilous epiphytes were inhabited by symbiotic ants belonging to the genera *Philidris*.

The lowland forest of Cat Tien National Park is located at the lowlands of Dong Nai province. The subequatorial monsoon climate of the National Park is characterized by two expressed seasons: a wet or rainy season (from May to October) and a dry one (from November to April) [45]. Cat Tien forests grow on relatively rich soils of volcanic origin, similar to bark-colored stratified Andosols [46]. A sample plot occupied about 1 ha around the 11°26′14″ N and 107°25′26″ E point. Epiphytes were collected from 25 individuals of phorophytes including 9 trees of the first canopy store were represented by *Lagerstroemia calyculata* (Lythraceae), *Afzelia xylocarpa,* and *Hopea odorata* (Dipterocarpaceae), and 16 trees from the lower layer were represented by *Aglaia* sp. (Meliaceae) and *Randia* sp. (Rubiaceae). The studied community of vascular epiphytes was represented by 40 species from 7 families of flowering plants and 3 families of ferns.

The mountain forest of Bidup Nui Ba National Park is located at Da Lat Plateau. The climate is characterized by the presence of expressed wet and dry seasons; however, it is much wetter and cooler than at neighboring plains of southern Vietnam. Materials were collected at Hon Giao mountain top (≈2000 m above sea level), in a shunted one-layered cloud forest, at the square of several hectares, around the 12°11′24″ N and 108°42′38″ E point. In total, epiphytes from 22 phorophyte individuals of *Castanopsis* sp., *Lithocarpus* sp. (Fagaceae), *Camellia* sp., *Schima* sp., *Ternstroemia* sp., *Eurya* sp. (Theaceae), *Magnolia* sp. (Magnoliaceae), *Pinus kesiya* (Pinaceae), and *Lyonia ovalifolia* (Ericaceae) were collected. The studied community of vascular epiphytes was represented by 23 species from 4 families of flowering plants and 3 families of ferns. Hemiparasites from *Viscum* sp. (Viscaceae) were also collected.

### 2.2. Samples Collection

Samples from each epiphyte, several fully developed adult leaves, were collected along with leaves of phorophytes on which they grew. Upper 1 cm of mineral soil at 1 m distance from the trunk of the phorophyte was also sampled. Leaves collected from one plant were mixed, so one sample represented one plant. We also collected hemiparasites from *Viscaceae* and *Loranthaceae* families, which mainly obtain nitrogen mainly from phorophytes [47,48]. Representatives of these groups are widely spread in open savanna-like forests of Phu Quoc Island, where they were collected by us. Additionally, we took non-vascular epiphytes (bryophytes and lichens) where they predominated.

While collecting epiphytes in the tall forest of Cat Tien, tree climbing was performed, and specimens were taken at the heights from 1.5 to 35 m. At Phu Quoc and Bidup, telescopic pole with crook at the top was used for collecting epiphytes at the heights from 1.5 to 6 m. In total, about 700 samples (including phorophytes, epiphytes, and soils under phorophytes) were collected and analyzed. Samples were dried immediately after collection at 60–70 °C.

### 2.3. Stable Isotope Analysis

Dried samples were pulverized in the Retsch MM 200 mill and wrapped in tin foil. Sample weight of plant material was about 1500 μg of soil up to 3000 μg. Isotopic composition of N and C was measured using Thermo Flash 1112 elementary analyzer and Thermo Delta V Plus isotopic mass-spectrometer at the Joint Usage Center at the A.N. Severtsov Institute of Ecology and Evolution, Moscow. The isotopic composition of N and C was expressed in the δ-notation relative to the international standard (atmospheric nitrogen and VPDB, respectively):

δX(‰) = [(R_sample_/R_standard_) − 1] × 1000,

where R is the ratio of the heavier isotope to the lighter isotope. Samples were analyzed with reference gas calibrated against the IAEA reference materials USGS 40 and USGS 41 (glutamic acid). The drift was corrected using the internal laboratory standards (casein, alfalfa). The standard deviations of the δ^15^N and δ^13^C values of reference materials (*n* = 8) were <0.2‰. Along with isotopic analyses, nitrogen and carbon contents (as mass %) and mass C/N ratio were determined in all samples.

### 2.4. Statistical Analysis

#### 2.4.1. General Patterns of δ^13^C in Leaves of Epiphytes

To reveal general patterns of δ^13^C in epiphyte leaves, we grouped all the species according to various characteristics:

(1) Functional group, which reflects both growth form and nutrient acquisition status: accumulative, succulent, mirmecophylous, hemiparasites, carnivorous, hemiepiphytes, and cryptogams (bryophytes and lichens);

(2) Taxonomic group: orchids, Araceae, Loranthaceae, Viscaceae, other dicots, ferns, bryophytes, and lichens;

(3) Synusia (the position along vertical profile of canopy layers is important for light perception): heliophytes, mid-stem epiphytes, and shading-tolerant plants (sciophytes). Here, we considered only autotrophic plants.

Since all the groups varied by the number of specimens in different communities, and sometimes this number was low, we used permutation tests for the statistical analysis. We ran two types of tests: (1) for between-community differences in δ^13^C of a single group, and (2) for within-community differences in δ^13^C between groups. If the group occurred in two communities only, or the community contained only two groups of species, the Approximative Fisher–Pitman Permutation Test was applied. In the case of more than two groups/communities, we used the Approximative k-sample Fisher–Pitman Permutation Test followed by pairwise permutation tests to reveal what groups differ. The tests were performed in *coin* and *rcompanion* R packages [49,50].

#### 2.4.2. Links of Epiphyte Leaves δ^13^C with Their Other Traits

To reveal links between epiphyte leaf δ^13^C and other leaf traits: leaf C and N content, C/N ratio, and δ^15^N, we calculated non-parametric Spearman correlation coefficients, as the distribution of the studied traits did not meet normality assumptions. Correlation coefficients were applied to the whole data set as well as to grouped data: functional groups, taxonomic groups, and synousia.

To visualize epiphyte traits, we ran the PCA (Principal Component Analysis) ordination with scaled data in the *vegan* package [51].

#### 2.4.3. Links of Epiphyte Leaf δ^13^C with Traits of Phorophytes

To compare mean δ^13^C values of epiphyte leaves with those of phorophytes by communities, we ran the Approximative General Independence Test, where pairs of phorophyte–epiphyte were concerned. If the same phorophyte carried several epiphytes, this plant was included in the analysis with the corresponding number of cases.

To reveal links of epiphyte leaf δ^13^C with traits of phorophytes, the mixed-effect linear models were fitted in the *nlme* package [52]. In the full model, the fixed effects were δ^13^C and δ^15^N, C and N content, and C/N ratio in the leaves of phorophytes. The random effect was an individual number of phorophytes because the same tree could support 1–13 sampling units (species) of epiphytes. The best model was selected in the course of automatic stepwise regression using the Akaike information criterion (AIC) and tested for the normality of residuals using quantile–quantile plots. The coefficient of determination for mixed linear models was calculated using the “r.squaredGLMM” function in the *MuMIn* package [53] using the algorithm of Nakagava and Schielzeth [54].

To comply with normality assumptions, the dependent variable δ^13^C was transformed by the constant addition (absolute minimum δ^13^C value plus 1), which allows the avoidance of negative values, and was log-transformed.

This analysis was run for all the epiphytes (427 samples from 137 phorophytes) and separately for different groups according to different traits and their combinations. The groups were based on the functional group, height of synousia affinity, and taxon group. In total, 10 combinations of the functional group—synousia—were distinguished: accumulative epiphytes—heliophytes (142 samples), mid-stem accumulative epiphytes (38 samples), accumulative epiphytes—sciophytes (19), mid-stem carnivorous plants (9), mid-stem cryptogams (38), hemiepiphyte heliophytes (11), parasites (36), myrmecophylous heliophytes (15), succulent heliophytes (85), and succulent sciophytes (34).

#### 2.4.4. Links of Leaf δ^13^C with Phorophyte Traits in Some Species

To estimate links of δ^13^C in the leaves of individual epiphyte species with the traits of phorophytes, we used ordinary least squares regressions. The selected species occurred in more than 10 samples: *Dischidia nummularia, Hoya* sp., *Loranthus* sp., *Pyrrosia adnascens* and *Pyrrosia longifolia*. The full model includes phorophyte traits (δ^13^C, δ^15^N, leaf C, leaf N, and C/N ratio). In the course of automatic stepwise regression, the non-significant factors were removed. The fitted models were tested for the residual normal distribution and variance inflation factor. One sample of *Loranthus* sp. and two samples of *Pyrrosia adnascens* with a δ^13^C value exceeded other conspecific values (less negative) and indicated CAM metabolism.

#### 2.4.5. General Picture of δ^13^C Distribution Trends

Following Messerschmid et al. [3], we conducted a mixture modeling analysis using the Rmixmod R package [55] to explore the complex and multimodal distribution of δ^13^C and δ^15^N values among epiphyte species, which could indicate differences in photosynthetic features and nitrogen sources. We determined the optimal number of clusters by fitting Gaussian mixture models with varying numbers of clusters (ranging from 1 to 10) and using the Bayesian Information Criterion (BIC). We repeated this process 10 times and used a maximum of 100,000 iterations for the EM algorithm of Rmixmod. We also ran the analysis 1000 times to ensure that we identified the most optimal number of clusters using Gaussian mixture models.

## 3. Results

### 3.1. General Patterns of δ^13^C Content in Leaves of Epiphytes

**Figure 1:**
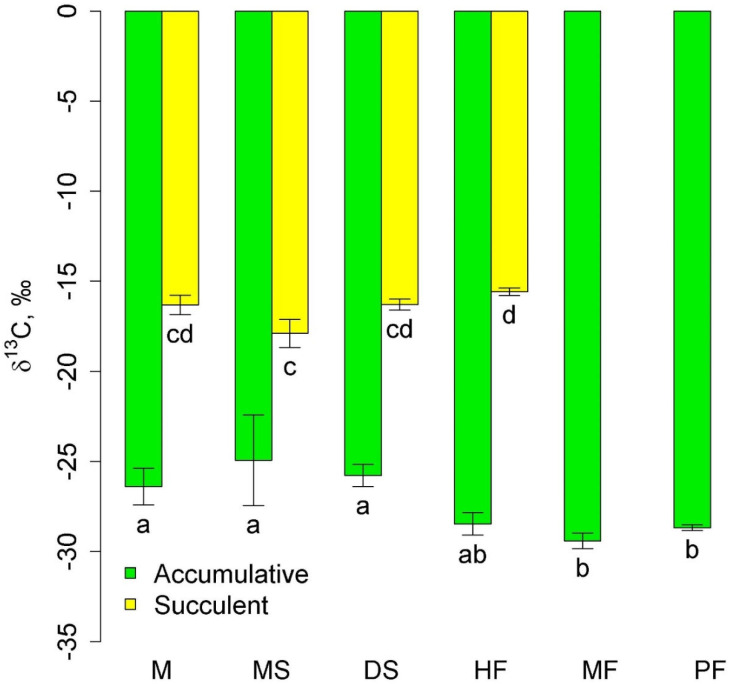
δ^13^C in accumulative and succulent epiphytes in different communities (mean and standard error). Phu Quoc: M—mangroves, MS—melaleuca savanna, and DS—dry savanna. Cat Tian: HF—high-stem forest. Bidoup: MF—mossy forest and PF—Pinus forest. Different letters show significantly different values (*p* < 0.05, Approximative Fisher–Pitman permutation test).

Two groups of epiphytes were most widely represented: accumulative epiphytes, which occurred in all communities, and succulent epiphytes, which were observed in all three lowland and mid-mountain communities. The δ^13^C values in succulent leaves were higher (less negative) than in accumulative epiphytes (Figure 1). The δ^13^C of accumulative epiphytes was significantly lower in the highland communities, moss forest and pine forest, than in the lowland communities. In succulents, the lowest δ^13^C values were in melaleuca savanna. Interestingly, δ^13^C of accumulative epiphytes tended to be lower, and succulent epiphytes tended to be higher in the mid-mountain tall grass forest compared to the lowland communities (Figure 1).

Semi-parasites were represented in two communities: dry savanna and melaleuca savanna, where they did not differ from each other in δ^13^C (*p* = 0.692).

**Figure 2:**
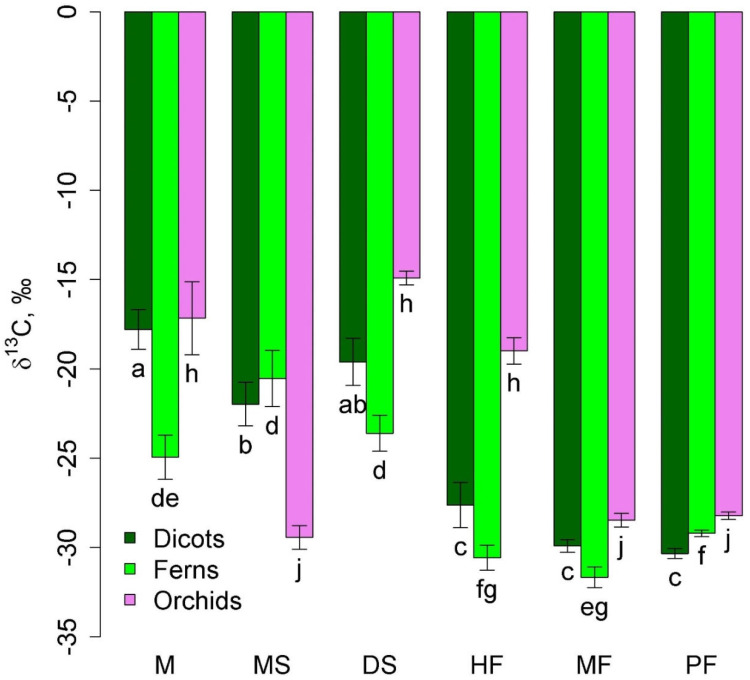
Leaf δ^13^C of dicots, orchids, and ferns in different communities (mean and standard error). Phu Quoc: M—mangroves, MS—melaleuca savanna, and DS—dry savanna. Cat Tian: HF—high-stem forest. Bidoup: MF—mossy forest and PF—Pinus forest. Different letters show significantly different values (*p* < 0.05, Approximative Fisher–Pitman permutation test).

Among taxonomic groups of epiphytes, orchids, ferns, and dicotyledons (excluding predatory and semi-parasitic plants) were widely represented. Ferns in lowland communities were characterized by less negative δ^13^C values than in montane forests (Figure 2). Two groups of communities were distinguished in terms of δ^13^C of orchids: mangrove, dry savanna, and tall-stem forest with less negative values and melaleuca savanna, moss forest, and pine forest with more negative values, where orchids were represented by accumulative epiphytes.

**Figure 3:**
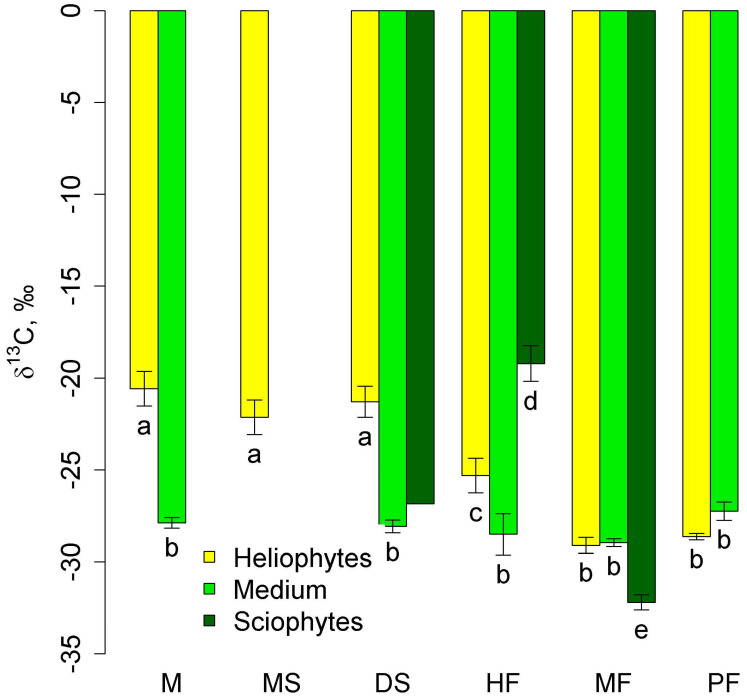
Leaf δ^13^C of autotrophic heliophytes, mid-stem epiphytes, and sciophytes in different communities (mean and standard error). Phu Quoc: M—mangroves, MS—melaleuca savanna, DS—dry savanna. Cat Tian: HF—high-stem forest. Bidoup: MF—mossy forest and PF—Pinus forest. Different letters show significantly different values (*p* < 0.05, Approximative Fisher–Pitman permutation test). Since in the dry savanna there is only one sample of sciophytes, the significance is not shown.

δ^13^C in mid-stem epiphytes did not differ between communities (Figure 3). Heliophytes in lowland communities had high (less negative) δ^13^C values that were significantly different from those of mid-stem epiphytes. With increasing altitude, they decreased significantly in the series of high-stem forests—moss forest and pine forest—and in the latter two communities, they did not differ from those of mid-stem epiphytes. Sciophytes were represented only in two types of communities—tall-stem forests and moss forests (in dry savanna, only one sample belonged to this group), and in the first community δ^13^C was less negative, and in the second community, more negative than in other groups (Figure 3).

### 3.2. Relationship between δ^13^C in Leaves of Epiphytes and Other Epiphyte Traits

The nature of the relationship between δ^13^C and other epiphyte traits was different between groups (Table 1). Thus, the general trend of decreasing δ^13^C in leaves with high carbon content was not evident at the level of ecobiomorphs but was true for heliophyte sinusia and for taxonomic groups of orchids and other dicotyledons. The negative relationship with nitrogen content observed for all samples was pronounced for all autotrophic plants from all sinusia, but in semiparasites (and loranthus as a separate taxonomic group), δ^13^C was positively correlated with nitrogen content. The overall negative relationship with δ^15^N at the level of most groups did not persist and was maintained only in the mid-stem epiphyte sinusia and in the group of other dicotyledons.

**Figure 4:**
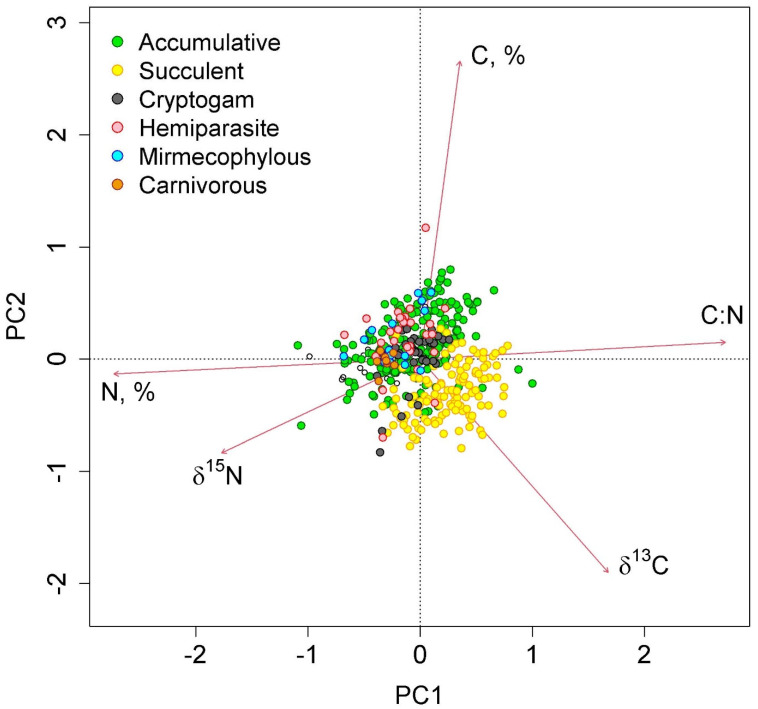
Ordination diagram of epiphyte traits (the results of the Principal Component Analysis). PC1 and PC2—the first and the second PCA axes (45.3 and 24.7% of variance explained, correspondingly). The colors show different functional groups.

**Figure 5:**
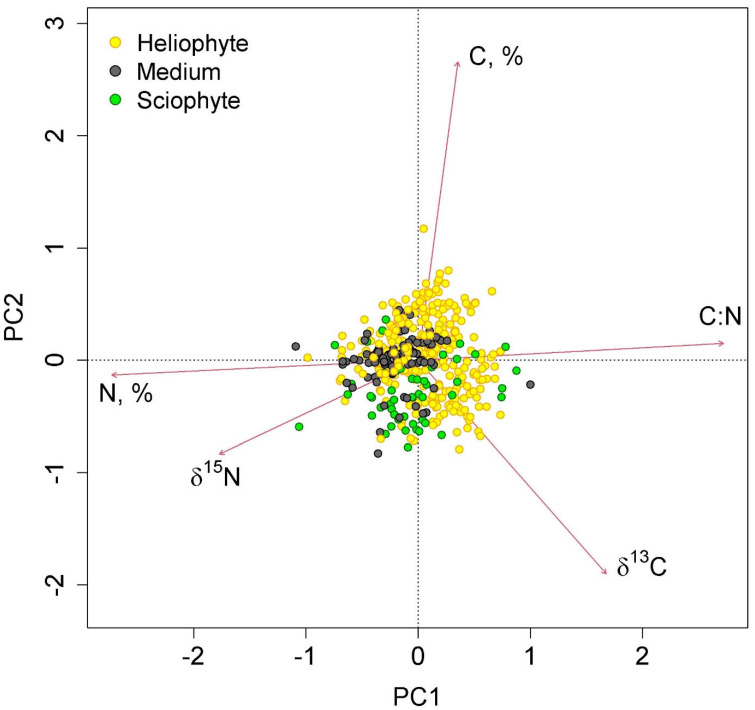
Ordination diagram of epiphyte traits (the results of the Principal Component Analysis). PC1 and PC2—the first and the second PCA axes (45.3 and 24.7% of variance explained, correspondingly). The colors show samples belonging to different canopy layers.

The first two axes of greatest variation explained 70.0% of the variance in the parameters of nitrogen and carbon content in epiphyte leaves (Figure 4 and Figure 5). The first axis was negatively correlated with nitrogen content, and the second axis was negatively correlated with carbon content.

### 3.3. Relationship between δ^13^C in Epiphyte Leaves and Phorophyte Traits

**Figure 6:**
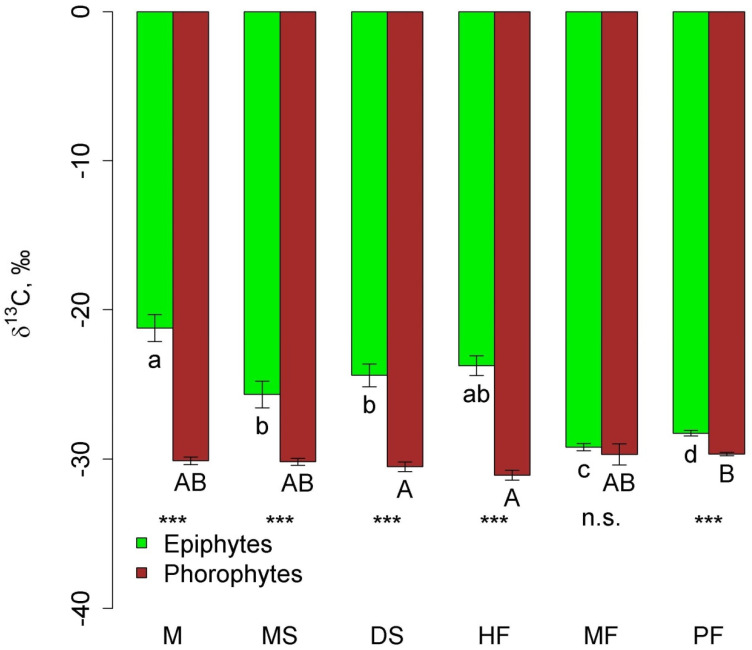
Leaf δ^13^C of epiphytes and their phorophytes in the different communities (mean and standard error. Phu Quoc: M—mangroves, MS—melaleuca savanna, and DS—dry savanna. Cat Tian: HF—high-stem forest. Bidoup: MF—mossy forest and PF—Pinus forest. Different letters show significant differences between communities (*p* < 0.05, Approximative Fisher–Pitman permutation test, lowercase letters—epiphytes, and uppercase letters—phorophytes). Asterisks show differences between epiphytes and phorophytes within the same community (Approximative General Independence Test): ***—*p* < 0.001 and n.s.—non-significant.

In all communities, except moss forest, δ^13^C in epiphyte leaves was significantly higher (less negative) than in phorophyte leaves (Figure 6). In general, δ^13^C in epiphytes in the mountain communities (pine forest and moss forest) was more negative compared to other communities due to the absence of succulents.

δ^13^C in the leaves of all epiphytes was negatively related to carbon percentage and δ^15^N in phorophyte leaves (Table 2). These traits explained 12.4% of the variation in the dependent variable. However, when looking at individual epiphyte groups, it appeared that relationships were possible with other traits.

A positive relationship between δ^13^C and the percentage of nitrogen in the phorophyte was shown for accumulative epiphytes—sciophytes, whereas no significant predictors were observed for other synusia and the group of accumulative epiphytes as a whole.

### 3.4. Relationship of δ^13^C in Leaves of Selected Epiphyte Species to Phorophyte Traits

Of the phorophyte traits, δ^15^N and carbon percentage were most often included in models explaining δ^13^C in leaves of the epiphytes that inhabit them. In *Pyrrosia adnascens*, these two parameters jointly explained 49.3% of the variation in δ^13^C. However, these relationships had different directions in different species. Thus, δ^13^C in *Dischidia nummularia* and *Loranthus* sp. was negatively correlated to δ^15^N of the phorophyte, while in *P. adnascens* and *P. longifolia* this relationship was positive (Table 3). Also, in the first two species the relationship with the percentage of carbon in the phorophyte was negative, in *P. adnascens* it was positive, and in *P. longifolia* it was absent.

## 4. Discussion

**Figure 7:**
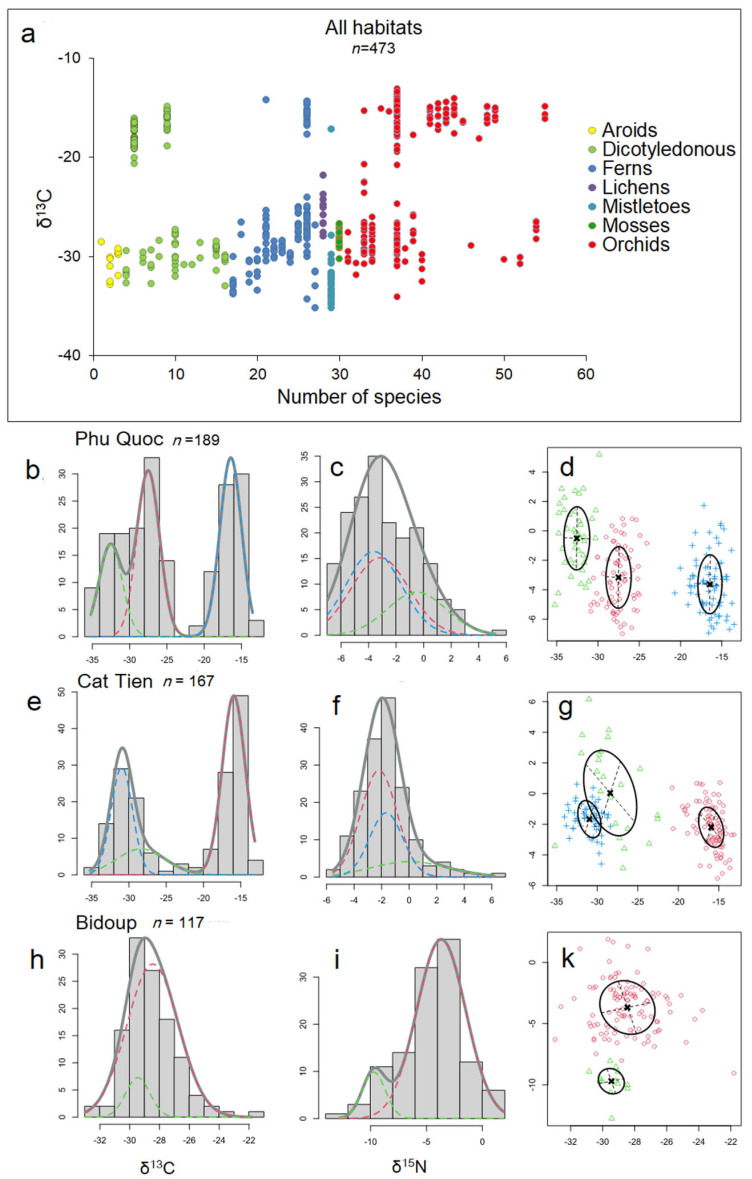
General trends in δ^13^C distribution: (**a**) bimodal distribution of carbon isotope signature values among epiphyte taxa. Each circle is the mean δ^13^C value of an epiphyte species, with color indicating taxa. The remaining slots show histograms illustrating the quantitative distribution of all δ^13^C and δ^15^N values of epiphytes separated by individual habitats. Gaussian curves in the histograms correspond to the best-fitting model obtained from the mixed modeling analysis. The colors of individual curves serve only for easier differentiation from other curves and have no particular significance. Frequency (ordinate) refers to the number of specimens, not taxa, and the total number of specimens (n) is indicated near the habitat name. Phu Quoc epiphytes: (**b**) histogram of δ^13^C values shows a trimodal distribution, with (**c**) unimodal distribution of δ^15^N, and the (**d**) relationship between δ^13^C (abscissa) and δ^15^N (ordinate) shows three clusters: indicated in blue for succulent epiphytes ferns and orchids (with strong CAM), in red for accumulative epiphytes (orchids, myrmecophiles, and ferns) and semi-parasites (weak CAM and C3), and in green for mosses and hyperparasites (C3). Cat Tien epiphytes: (**e**) histogram of δ^13^C values shows bimodal distribution, (**f**) unimodal distribution of δ^15^N, and the (**g**) relationship between δ^13^C and δ^15^N shows three clusters: red—succulent epiphytes, mainly orchids (strong CAM), green—accumulative epiphytes and semi-epiphytes, mainly orchids (C3 and weak CAM), and blue—accumulative epiphytes, ferns, dicotyledons, and aroids (C3). Bidup: (**h**) histogram of δ^13^C values shows unimodal distribution and (**i**) bimodal distribution of δ^15^N, and the (**k**) relationship between δ^13^C and δ^15^N shows two clusters: red—accumulative, carnivorous, and other epiphytes, mainly orchids (C3) and green—accumulative epiphytic ferns (C3).

Our study revealed both trends similar to those described in the literature and contradicted them, which can be explained by a different set of biomorphological features of epiphytes from tropical Asia, compared to Neotropis epiphytes, where the majority of such work has been conducted. The general trends of δ^13^C distribution on the one hand resemble those traditionally described: we see that there is a bimodal distribution of δ^13^C within epiphytic plant taxa (Figure 7a), and on the other hand, the study of Gaussian distributions via mixture modeling using the Rmixmod R package [55] showed a more complex pattern reminiscent of that recently described by Messerschmid et al. [3]. Thus, for Phu Quoc epiphytes: the histogram of δ^13^C values shows a trimodal distribution (Figure 7b), for Cat Tien epiphytes shows a bimodal distribution (Figure 7e), and for Bidup shows a unimodal distribution (Figure 7h), with a unimodal distribution of δ^15^N in all cases (Figure 7c,f,i).

More interesting is the comparison of the δ^13^C and δ^15^N relationship using the aforementioned method: we see, in general, the formation of several clusters, where succulent epiphytes gravitate more toward strong CAM at more negative values of δ^15^N, and accumulative epiphytes gravitate toward weak CAM or C3 at more positive values of δ1^5^N (Figure 7d,g,k). This is in good correspondence with Stewart et al. [56] who, based on δ^15^N values and total nitrogen concentrations, distinguished two groups of epiphytes—one with low total nitrogen and low ^15^N as well as one with higher total nitrogen and δ^15^N. However, no clear correlation between δ^13^C and δ^15^N was found [25] and appears not to be meaningful in the context of methods that assess correlation (Table 1).

We also found, as was suggested previously (1), that δ^13^C depends not only on the tier localization (synusia) and taxonomic group of the epiphytic plants but also on their life form. For all epiphyte samples, negative correlation of δ^13^C values has been shown with the percentage of carbon and δ^15^N in their leaves (Table 1). At the same time, we observed a decreasing trend of δ^13^C with altitude (Figure 1 and Figure 2) in accordance with that described in the literature [4,25]. That is a trend of δ^13^C increasing with tree height [9,16,27,28]. It is complicated by the presence of a group of sciophyt epiphytes (orchids) in the Cat Tien Forest growing at human height level that had a pronounced CAM carbon signature. These are the very small orchids *Grosourdya appendiculata* and *Dendrobium oligophyllum*, as well as some miniature representatives of the genera *Gastrochilus* and *Phalaenopsis*. No CAM epiphytes have been reported for tropical biomes of America under the canopy of a tiered forest for such heights [9,27]. We previously investigated them, assuming a significant involvement of mycorrhizal myxotrophy “weighting” carbon, but no confirmation was found [43]. We also investigated their respiration and the diurnal course of acidity. Both measurements were made at the end of the wet season (when the partially deciduous forest is maximally green). Both the diurnal course of respiration and acidity suggest rather facultative CAM, which contradicts their pronounced CAM signature. It remains to be assumed that in this case, CAM serves not as much as an adaptation to lack of moisture but as an evolutionary path of miniaturization, allowing these plants to remain inconspicuous and thrive in a tier where large animals or humans are very active. The less “carbon-efficient” CAM compared to C3 in more shaded conditions allows plants to drastically reduce their size. They grow actively precisely in the drier and brighter season, when the main carbon masses of leaf tissues follow down in the form of the cellulose of cell walls, while in the wet season, they only flower and fruit. As far as we know, such a strategy of CAM as adaptation to miniaturization in the lower tier has not been described previously.

We also hypothesized that (2) δ^13^C would be significantly correlated with phorophyte traits due to the “forest canopy effect”. For all epiphyte samples, δ^13^C was negatively correlated with carbon percentage and δ^15^N in phorophyte leaves (Table 2). These correlations were not observed in the groups of accretive epiphytes and sciophytes, but there was a significant negative relationship with the percentage of nitrogen, whereas for the group of accretive epiphyte-sciophytes, there was a significant positive relationship with nitrogen content in phorophyte leaves (and it was not significant in accretive epiphyte-heliophytes). The fact that we did not observe a relationship with δ^13^C in phorophytes (or rather, we observed it only for semi-epiphytes) is most likely due to the specificity of epiphyte sampling strategy. We did not take leaves of a phorophyte duplicating the altitudinal localization of the phorophyte but took a single collected sample from a tree. But we do observe correlations with C% and δ^15^N of phorophytes determined by some third factor (Table 2). This factor may be the more broadly understood by the “forest canopy effect”, which needs to be conceptualized not only as vertical stratification of carbon isotopes but also the overall respiration-related effects of changes in carbon and nitrogen content in the tree canopy. Evidence for a fairly unambiguous canopy effect on epiphytes is provided by comparing *Dischidia nummularia* (a typical epiphyte with CAM) and *Loranthus* sp. (a typical semi-parasite) (Table 3). Both species show a striking similarity of association with the tree for both carbon and nitrogen parameters. This is difficult to explain in terms of traditional views of epiphytism as the absence of parasitic relationships with the host, unless one takes the “forest canopy effect” more broadly: as it is the causal changes in carbon parameters (and associated nitrogen through specificity of mineral nutrition) in the crowns of phorophytes and the semi-parasites that inhabit them (which is understandable), as well as epiphytes (which is still poorly understood).

Finally, within (3) of the last hypothesis, we hypothesized that δ^15^N cannot be a predictor of δ^13^C changes in epiphytes due to the presence of the δ^15^N correlation between phorophytes and epiphytes that we described earlier [57], which can be explained by the edificatory role of epiphytes. Even Stewart et al. [56] distinguished two groups of epiphytes, one with low total N and low δ^15^N, and one with higher total N and δ^15^N. Clearly, the first trend is an adaptation at CAM (a group that is typified as succulent epiphytes within this work) and the second at C3 and is associated with a large water supply and diversified food sources (accumulative epiphytes within this work). Judging by this scheme, δ^13^C should correlate with δ^15^N, which in reality we do not observe, in all but two categories, and the correlation was not significant, although the relationship with gross nitrogen is more obvious (Table 1). Although as stated above, when we examine the Gaussian distributions via mixture modeling using the Rmixmod R package, we see the group formation (Figure 7d,g,k) resembling that predicted by Stewart et al. [56]. But it is possible to look at this problem from another angle. It is possible that epiphytic colonization has some influence on the nitrogen nutrition of the phorophyte. The ecosystem role of nitrogen deposited in epiphytic material (EM) is difficult to assess. Current estimates suggest that it is quite low, if not negligible. For example, in Chinese montane forests, epiphytic nitrogen was 37.9 ± 9.0 kg/ha, and the total EM mass was 2261 ± 537 kg/ha [58]. On the other hand, the epiphytic community can exert influence through a large number of biotic relationships, such as with ants and other invertebrates, small vertebrates, fungi, and cyanobacteria. We can also speculate that the ecosystem contribution of epiphytes to N balance is not in gross N accumulation but in buffering the N cycle in crowns and preventing N leaching. This may explain our observed absence of a predicator role of ^15^N in CAM distribution, expressed in the absence of correlation between δ^13^C and epiphytes. For example, almost all CAM epiphytes observed in low-stemmed sparse formations of Phu Quoc are inhabited by mutualistic ants of the genus *Philidris* in 90% of cases [44], and the correlation between δ^15^N of epiphytes and trees was maximal in these formations [57]. It remains to be assumed that CAM epiphytes in this situation not so much receive nitrogen nutrition from these ants, but by giving them shelter, they themselves indirectly influence the nitrogen nutrition of trees, which receive nitrogen from flushes during rainfalls. Thus, epiphytes implement a kind of edificatory function. Thus, our study shows that parameters marking CAM in epiphytes are in a rather complex relationship both with each other and with tree parameters. This suggests the need to change the focus of the whole problem.

In a study of over 1000 orchid species from Panama and Costa Rica, Silvera et al. [12] found δ^13^C values indicative of CAM (δ^13^C > −20‰) in about 10% of all epiphytic species, while CAM was clearly exceptional among terrestrial species with 4 out of 121 species (3%). Thus, CAM was indeed more common among epiphytic species than terrestrial species, but the percentage of species with CAM was not very high. A more recent study of Colombian orchids by Torres-Morales et al. [14] found a similar figure for epiphytic species but more CAM species among terrestrial species: the proportion of CAM species was 9.5% in epiphytes (76/805 species) and 7.3% in terrestrial species (19/260 species). We are not aware of such data for Tropical Asia, but we can hardly expect other trends. This picture is usually interpreted in terms of adaptations to water retaining, since water scarcity is considered to be the main limiting factor for epiphytes [24], but our research shows that the picture may be much more complex.

## 5. Conclusions and Future Work

As part of our study, we found that the carbon isotopic signature of epiphytes is related to nitrogen content in both epiphytes and their host trees. In all communities, except for the moss forest, δ^13^C in epiphyte leaves was significantly higher (less negative) than in phorophyte leaves. In general, δ^13^C in epiphytes in mountain communities (pine forest and moss biotopes) was more negative than in other communities due to the absence of succulents with CAM. δ^13^C in the leaves of all epiphytes was negatively related to the percentage of carbon and δ^15^N in the leaves of the phorophyte. These features explained 12.4% of the variation in the dependent variable. However, when considering individual groups of epiphytes, it turned out that relationships with other characters are possible. When considering the Gaussian distributions of δ^13^C via the method of modeling mixtures, and we observe unimodal, bimodal, and trimodal distributions.

It should be taken into account that epiphytes are a unique biological object—which grow under natural hydroponic conditions. And the ways of supplying even the most common elements of mineral nutrition are in many respects poorly understood. For terrestrial plants the presence of such important K, Ca, Mg, and Na for photosynthesis and in general for metabolism nutrients is more or less stable due to their presence in silicate lattices of minerals composing terrestrial soils and further transferring to the soil absorbing complex. The ways of obtaining and influencing their concentrations on CAM in epiphytes are not known at all. But the coastal formations of Phu Quoc, where epiphytes with CAM flourish, are exposed to the influence of “salty” impulverization breezes from the sea and are most likely not deficient in these elements, while the light pine forests of Bidup, where there is a lot of sun and wind, and epiphytes quite show CAM morphology, and we do not observe them with carbon isotope signature. This may demonstrate perhaps not of an excess of water supply (which is not present in the pine forests) but of a deficiency of K, Ca, Mg, and Na in the very oligotrophic conditions of mountain epiphyte nutrition. To obtain a global picture of CAM in epiphytes, we should study the nature of δ^13^C connection with non-volatile elements of mineral nutrition, whose entry into epiphyte tissues is not clear and whose movement in crowns is very poorly investigated.

Thus, we suggest that further research into the carbon isotopic signature of epiphyte tissues should be carried out in the context of their relationship with a number of parameters encoding ecophysiological features; it is also desirable to study functional diversity based on the studied traits, which can provide insight into niche differentiation and ecosystem functioning.

## Figures and Tables

**Table 1:** Relationships between leaf δ^13^C and other traits of epiphytes in different functional, taxonomic, and ecological groups. Spearman correlation coefficients and *p*-values are shown. *—*p* < 0.05; **—*p* < 0.01; ***—*p* < 0.001; and n.s.—non-significant. The sum of sample numbers by groups can be less than the total sum because we did not run a separate analysis for groups with a small number of samples. One species can be represented by several samples.

Group	Number of Samples	C, %	N, %	C/N	δ^15^N
Total	427	−0.201 ***	−0.355 ***	0.319 ***	−0.232 ***
**Functional group**					
Accumulative epiphytes	199	−0.011 ^n.s.^	−0.398 ***	0.371 ***	−0.094 ^n.s.^
Succulent epiphytes	119	0.081 ^n.s.^	0.237 **	−0.212 *	−0.040 ^n.s.^
Hemiparasites	36	−0.021 ^n.s.^	0.518 **	−0.495 **	−0.046 ^n.s.^
Mirmecophylous epiphytes	15	−0.271 ^n.s.^	0.186 ^n.s.^	−0.218 ^n.s.^	0.036 ^n.s.^
**Taxonomic group**					
Bryophytes	28	−0.199 ^n.s.^	0.220 ^n.s.^	−0.260 ^n.s.^	−0.031 ^n.s.^
Ferns	114	−0.044 ^n.s.^	−0.469 ***	0.452 ***	−0.073 ^n.s.^
Loranthaceae	27	0.081 ^n.s.^	0.538 **	−0.509 **	−0.059 ^n.s.^
Other dicots	90	−0.472 ***	−0.670 ***	0.609 ***	−0.257 *
Orchids	138	−0.460 ***	−0.171 *	0.089 ^n.s.^	0.064 ^n.s.^
**Canopy layer**					
Heliophytes	253	−0.387 ***	−0.492 ***	0.430 ***	0.032 ^n.s.^
Mid-stem epiphytes	85	0.151 ^n.s.^	−0.498 ***	0.468 ***	−0.407 ***
Sciophytes	53	−0.020 ^n.s.^	−0.340 *	0.309 *	−0.215 ^n.s.^

**Table 2:** Relationship of leaf δ^13^C in epiphytes with traits of phorophytes. n—number of samples (epiphytes). N—number of phorophytes. p—significance level. R^2^m—coefficient of determination for fixed effects. R^2^c—coefficient of determination for fixed and random effects. Only significant models are shown.

Epiphytes	n	N	Predictors: The Traits of Phorophytes	Effect	χ^2^	p	R^2^m	R^2^c
All epiphytes	427	137	C, % δ ^15^N	– –	43.94 5.96	<0.001 0.015	0.124	0.213
Accumulative epiphytes-sciophytes	19	11	N, %	+	10.19	0.001	0.361	0.361
Hemiepiphytes-heliophytes	11	9	δ ^13^C	+	6.93	0.008	0.410	0.410

**Table 3:** Relationships of leaf δ^13^C in individual species of epiphytes with the traits of phorophytes. df—degrees of freedom. R^2^—determination coefficient. p—significance level for the model. B—regression slope. StE B—standard error of the regression slope. P—significance level for a single factor. *—*p* < 0.05; **—*p* < 0.01; ***—*p* < 0.001.

Species	df	R^2^	p	Predictors: The Traits of Phorophytes	B	StE B	P
*Dischidia nummularia*	2, 26	0.333	0.005	δ^15^N C, %	−0.220 −0.084	0.062 0.058	0.002 ** 0.161
*Hoya* sp.	15			нeт			
*Loranthus* sp.	3, 18	0.467	0.009	C, % C/N δ^15^N	−0.228 −0.056 −0.373	0.103 0.025 0.177	0.039 * 0.039 * 0.049 *
*Pyrrosia adnascens*	2, 20	0.493	0.001	δ^15^N C, %	0.284 0.390	0.069 0.125	<0.001 *** 0.006 **
*Pyrrosia longifolia*	1, 15	0.292	0.025	δ^15^N	0.168	0.068	0.025 *

## Data Availability

Not applicable.
